# Fiber-Optic Sensors for Measurements of Torsion, Twist and Rotation: A Review †

**DOI:** 10.3390/s17030443

**Published:** 2017-02-23

**Authors:** Vedran Budinski, Denis Donlagic

**Affiliations:** Laboratory for Electro Optics and Sensor Systems (LEOSS), Faculty of Electrical Engineering and Computer Science, University of Maribor, Smetanova ulica 17, Maribor 2000, Slovenia; vedran.budinski@um.si

**Keywords:** fiber optic sensors, twist sensors, rotation sensors, circular birefringence, linear birefringence, FBG, tilted FBG, long period FBG, polarization in optical fibers, optical fibers

## Abstract

Optical measurement of mechanical parameters is gaining significant commercial interest in different industry sectors. Torsion, twist and rotation are among the very frequently measured mechanical parameters. Recently, twist/torsion/rotation sensors have become a topic of intense fiber-optic sensor research. Various sensing concepts have been reported. Many of those have different properties and performances, and many of them still need to be proven in out-of-the laboratory use. This paper provides an overview of basic approaches and a review of current state-of-the-art in fiber optic sensors for measurements of torsion, twist and/or rotation.

## 1. Introduction

Measurement of mechanical parameters through application of optical fibers is gaining significant commercial interest in different industry sectors. This interest is driven by a unique set of properties provided by fiber-optic sensor technology, and maturing opto-electronic signal interrogation systems that have become available over the past decade. Among the best-developed fiber-optic sensor technologies for measurements of mechanical parameters are probably Fiber Bragg Gratings (FBGs) and Fabry-Perot Interferometer (FPI) sensors. While FPIs dominate optical pressure sensing applications, FBGs are, in their essence, strain sensors, and can be configured in a wide range of sensing systems where measured mechanical quantity can be correlated unambiguously with the strain as, for example, in the case of force, load, stress, and similar measurements. None of these commercially successful technologies, however, addresses measurements of rotation, twist and torsion adequately, which are among the most frequently measured mechanical parameters in a wide range of industrial sectors. Measurements of twist/rotation have, thus, recently been topics of intense research within the fiber-optic sensor community. Furthermore, many applications in rotation/twist/torsion sensing cannot tolerate the high-cost of the measurement systems. Thus, successful introduction of these new types of sensors will depend on balanced development of both sensing concepts and accompanying signal interrogation. This review article provides a review of the basic principles and current state-of-the-art in fiber optic rotation, twist and torsion sensors.

Early work in the field of fiber-based twist/rotation sensors appeared in late 1980s, together with research in other subfields of the then emerging fiber-optic-sensor technology. These early solutions utilized either extrinsic approaches, for example, by inserting retardation plates between two collimated fibers [1], or intrinsic approaches using geometric effect of polarization rotation [2] and mode interface in highly multimode fibers [3]. The research in the field of twist/rotation/torsion, however, remained low until the early 2000s, when the number of publications in this field started to see a steady increase.

As of today, available sensor concept can be classified roughly (and not fully unambiguously) according to their physical principle of operation into the following groups/categories:

(A) Sensors based on the twist induced birefringence modulation in optical fibers.
(B) Sensors based on modulation of radial shear stress distribution.
(C) Twist/rotation sensors based on the spatial E-field displacement.
(D) Other twist/rotation sensors.

Each of these large categories can be divided further into sub-categories, as attempted with the descriptions below.

## 2. Sensors Based on the Twist Induced Birefringence Modulation in Optical Fibers

This class of sensors utilizes measurements of changes in a sensing fiber’s birefringence that are caused by fiber twist. Twist induced birefringence has been a topic of investigation since the early era of fibers. Fundamental investigations of this phenomenon date back to the late 1970s, early 1980s [4,5] and have continued up to recent years [6,7,8,9]. Twisting of a single-mode fiber causes two effects:

(a) Appearance of circular birefringence (which is also demonstrated as an appearance of optical activity).
(b) Modulation of linear birefringence, if linear birefringence pre-exists in the sensing fiber.

### 2.1. Twist Induced Circular Birefringence and its Use in Twist/Torsion Sensors

Torsional twisting of an optical fiber induces a torsional stress within the same fiber that results in a torsional elasto-optic effect which, further, leads to the appearance of circular birefringence [4,5,7]. The twist induced circular birefringence ∆*n_c_* is proportional to the elasto-optic coefficient *g* of the silica and the twist rate τ imposed on the fiber, e.g., ∆*n_c_* ∝ *g*τ. Thus, when a perfectly circular-symmetric and linear birefringence free fiber is exposed to a twist/rotation, it behaves almost as a perfect circularly birefringent (optically active) transmission medium. If a short section of twisted fiber (but otherwise straight fiber) is excited by linearly polarized light, the linear polarization of the launched light will be preserved; however, the polarization plane will be rotated along the fiber length (similarly as in an optical active medium). This polarization plane rotation corresponds to about 0.069° per degree of fiber’s mechanical rotation/twist around its z-axis [4]. Thus, the polarization plane rotation induced by mechanical fiber twist is relatively small when compared to mechanical fiber rotation around the z-axis. Twist induced circular birefringence is also temperature dependent, with a temperature coefficient of about 9.6 × 10^−5^ K^−1^ [4].

**Figure 1:**
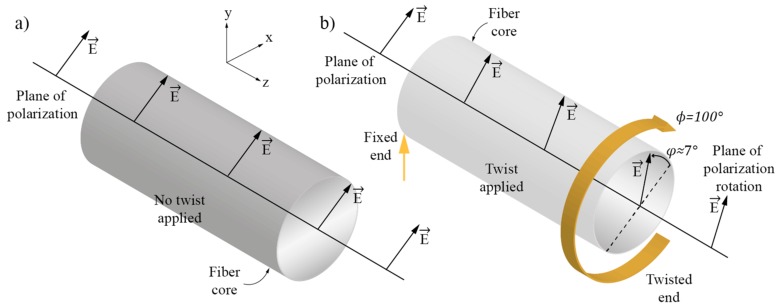
(**a**) Polarization plane and fiber direction when fiber is not twisted and (**b**) When fiber is twisted through 100°, the polarization plane is rotated through 7° in the opposite direction.

For example, if polarized light is launched into a single-mode fiber on one fixed end, and if the same fiber is twisted around its z-axis, the polarization plane at the output of the fiber will be rooted with a rate of 0.069° per degree of the fiber’s mechanical rotation (Figure 1). This manifestation of polarization rotation in single-mode fibers was investigated in different references [4,7]. The direct observation of this polarization rotation would require a rotary joint or similar mechanical free-space coupling between the twisted and lead-out fiber, which would probably be impractical for sensor implantation.

**Figure 2:**
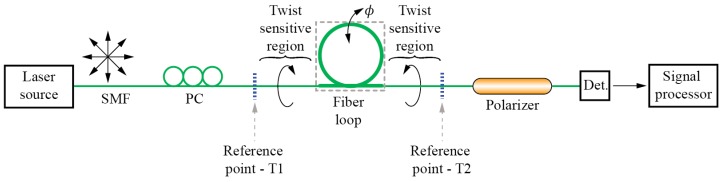
Fiber loop sensor system for angular measurements.

There are, however, reports that utilize direct measurements of a fiber’s circular birefringence induced by fiber twisting. An approach like this was, for example, proposed in [10] where an FBG was written into an SMF fiber which was exposed to twist/rotation. Measurement of the wavelength deepened Polarization Dependent Loss (PDL) around the FBG’s central wavelength revealed two distinctive spectral peaks in the PDL spectrum, with an amplitude ratio that was twist/rotation dependent. An interesting approach was presented in [11] where a fiber-loop was inserted in-between two fixed fiber points, while the loop plane was rotated around the fiber axis. These twisted fiber sections between the fixation points and the fiber loop and caused the appearance of circular birefringence proportional to the loop plane rotation angle, while the loop itself acted as linear birefringent retarder (due to the bend-induced linear birefringence). This created a distinctive variation of polarization states at the output of the sensor that was correlated unambiguously to the loop plane rotation (Figure 2).

### 2.2. Sensors Based on Twist-Induced Linear Birefringence Modulation

**Figure 3:**
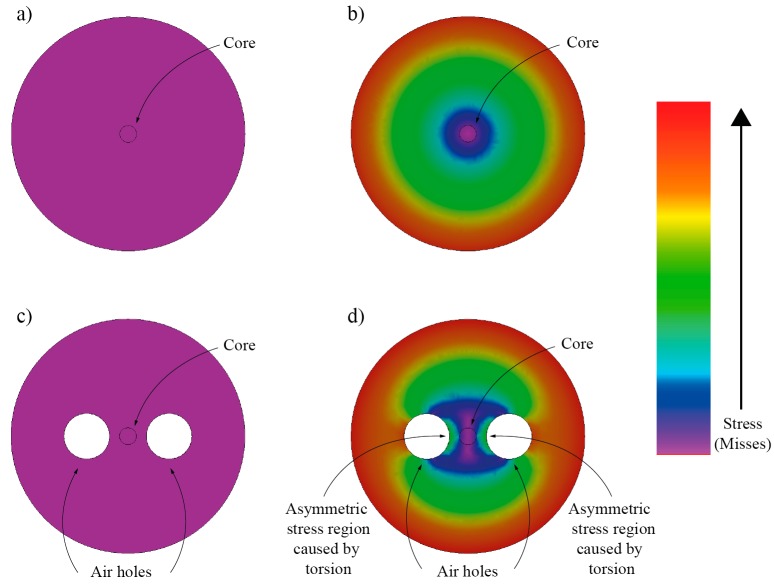
Finite element method simulation of a total stress distribution in a circularly symmetric fiber and circular non-symmetric fiber (containing two hollow regions): (**a**,**c**) show stress distribution when no twist is applied; (**b**,**d**) show the case when fibers are exposed to the twist. In case of the twisted fiber with side holes, a non-circularly symmetric stress build-up around the core can be observed, leading to linear birefringence modulation.

When a linear-birefringent single-mode optical fiber is twisted, its linear birefringence becomes dependent on the fiber’s twist rate (Figure 3). This is due to the circular non-symmetry of the linearly-birefringent fiber, which gives rise to different magnitudes of photo-elastic effects along the fast and the slow axes. The twist induced change in linear birefringence is proportional to the twist magnitude and fiber’s circular non-symmetry (and, consequently, to the fiber’s initial birefringence). Hence, this effect is only present in optical fibers that exhibit initial/intrinsic linear birefringence (Figure 3d). Twisting of a perfectly circuitry symmetric/homogenous optical fiber will not cause the appearance of linear birefringence within the same fiber (Figure 3b). It is, thus, the presence of the fiber’s circular non-symmetry in the fiber that gives rise to a measurement effect that can be observed through observation of changes in the fiber’s linear birefringence caused by torsional twist/rotation.

In general, three distinctive situations can occur: (1) When intrinsic linear birefringence is very low as, for example, in modern Telecom Singlemode Fiber (SMF), polarization effects related to the modulation of the linear birefringence are insignificant and circular birefringence related effects prevail (i.e., optical activity); (2) When fibers with low, but non-negligible, intrinsic linear-birefringence are twisted, the appearance of circular birefringence combines with linear birefringence modulation, which leads to the appearance of elliptical birefringence and complex polarization evolution along the fiber length [8,9]. Examples like this are some (highly) doped, but otherwise circular fibers when exposed to twisting; (3) When the fiber is Highly Linearly Birefringent (HLB), twist-induced effects related to linear birefringence modulation dominate, and effects related to the presence of circular birefringence become insignificant. The latter can also be explained by the strong splitting of polarization modes in HLB fibers, which “guarantees” independent and mode-coupling-free propagation of both linearly polarized modes within HLB fiber. Thus, high linear birefringence prevents any twist induced polarization plane rotation and, when only one linearly polarized mode is excited initially, spatial orientation of the E-field vector will follow one of the fiber’s birefringent axes, even when the fiber is exposed to a strong twist/rotation as demonstrated in [7].

Thus, it is not surprising that HLB fibers are the preferred choice for building birefringence modulation based twist/rotation sensors. The drawback of using HBL fibers is, however, in the relatively large temperature dependence of linear birefringence, which is an intrinsic characteristic of the stress induced birefringence found in conventional HLB-fibers, like PANDA or Bow-Tie fibers. Thus, many attempts have been made to design twist/torsion sensors using HLB Photonics Crystal Fibers (PCF) or fibers with structures that involve air holes for achieving high birefringence, as these fibers exhibit considerably lower linear birefringence dependence on the temperature.

**Figure 4:**
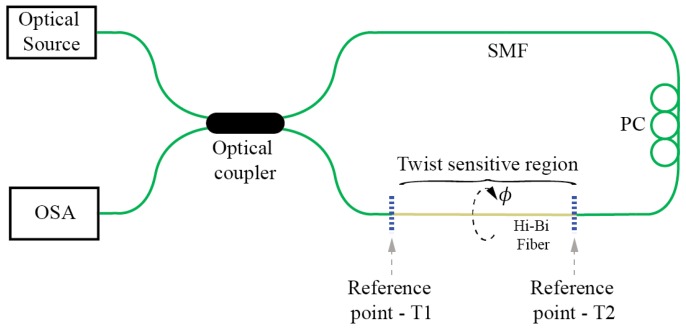
Twist/rotation sensor employing a Sagnac interferometer.

Known approaches for the measurements of linear birefringence variations in optical fibers usually involve interferometric methods. Among these methods, Sagnac interferometers/loop mirrors seems to dominate. The Sagnac interferometer/loop mirror provides a straightforward way for measurement of a fiber’s birefringence. When a section of single-mode fiber in the loop is replaced by linearly birefringent fiber, the birefringent fiber induces a wavelength dependent nonreciprocal phase shift among waves propagating in opposite directions within the fiber loop [12,13] as illustrated in Figure 4. This results in the loop’s distinctive spectral fringe pattern, with period and phase dependent on the inserted fiber’s birefringence and length, which makes spectral interrogation a preferred choice for extraction of a sensor signal. The spectral fringe contrast is maximum when both birefringent modes are excited equally, thus, some sort of polarization control is usually needed within the loop.

Sagnac interferometers/loop mirrors were applied successfully to a variety of different twist/rotation sensor designs that utilized conventional fibers or PCF with low or high birefringence. When low birefringence fibers are utilized in twist/rotation sensor designs, these sensing fibers must possess sufficient initial birefringence to obtain the desired measurement effect. Thus, when an SMF type of fiber is utilized, linear birefringence must be induced through external fiber perturbation, for example, side pressure, as described in [14]. PCF fibers usually possess intrinsically sufficient linear birefringence that provides an opportunity for twist/rotation sensing as, for example, described in [15]. These sensors provide lower sensitivities than sensors designed with HLB fibers, however, their main advantage is in their low intrinsic temperature sensitivity. Twist sensors configured in Sagnac loops that base on HLB fibers can, however, provide high twist/rotation sensitivities as demonstrated for sensors with elliptical core fibers [16], high-birefringent fibers with large side air holes [17] and side-leakage PCF [18,19]. Most of these approaches, however, exhibit non-negligible temperature dependence and require proper temperature compensation schemes (some solutions possess this capability intrinsically). Proper PCF design, however, also allows for creation of the Sagnac loop based twist/rotation sensors with very low intrinsic temperature sensitivities as described in [20].

**Figure 5:**
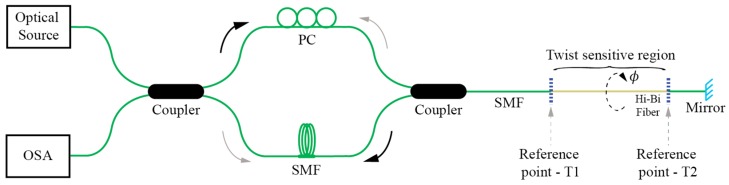
Twist sensor employing a loop mirror configuration with an output port probe.

While Signac interferometers/loop mirrors present a very convenient and versatile way for twist/rotation sensing, perhaps their main disadvantage comes from the need to access the sensor fiber from both sides and locate the entire fiber loop at the measurement site. This requirement might, importantly, compromise the practicality of Sagnac sensor setup, as it increases the size and complicates the sensor’s packing. A possible solution to overcome this limitation was presented in [21], where an additional coupler was inserted into the loop mirror to obtain a “linear” twist/rotation sensor configuration [22] using only one lead-in fiber (Figure 5). Another approach described in [23] attempted to make the entire Sagnac loop sensor more practical by considerable miniaturization of its size. In this instance, the fiber’s loop coupler was employed simultaneously as a coupler and birefringent sensing fiber section.

Sagnac interferometers/loop mirrors can also be equipped with active fibers and an optical pumping system to form lasers that generate output frequency that is dependent on torsional twist/rotation of the sensing section as shown in [24,25].

**Figure 6:**
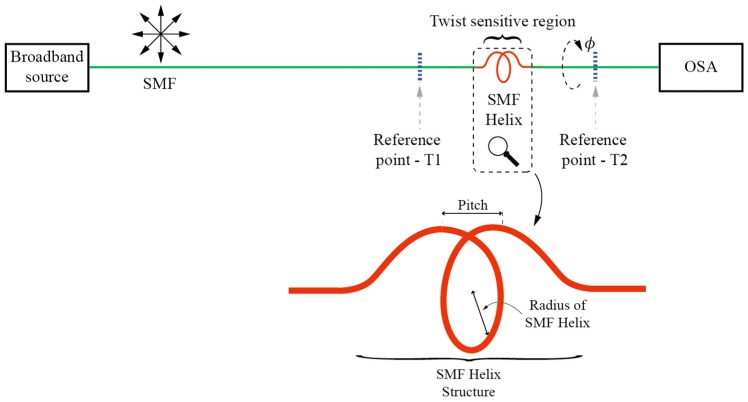
Modal MZI twist sensor experimental setup and illustration of the fabricated SMF Helix.

Besides Sagnac interferometers other interferometric methods can also be used efficiently to build HBL fiber based twist/rotation sensors. Excitation of both polarization modes in an HLB sensing single-mode fiber, for example, by aligning the polarization of the input E-field at a 45° angle relative to the principal axis of the HLB fiber, leads to the interference of both polarization modes along the fiber. This interference depends on the fiber’s birefringence, and information on the twist/rotation can be obtained when the polarization is analyzed at the sensing fiber’s output, (usually by adding a linear polarizer). A compact and elegant design of this type of sensor was presented in [26] where twist induced birefringence modulation of a PCF was measured through the interference of both polarization modes, while in-line fiber polarizers, required for initial controlled excitation of both polarization modes and for output analysis, were obtained by partial collapse of the PCF structure through application of a CO_2_ laser. Another example of this approach, also using PCF, was presented in [27]. A curious twist sensitive birefringence modulation sensor is described in [28], where a relatively large helix made out of a SMF is fabricated by a flame-heat treatment (Figure 6). Curved/helix fiber possess frozen-in birefringence and when a linearly polarized light is launched through the SMF helix, two orthogonally polarized modes are excited with different modal refractive indices. After passing through the helical section, those two modes recombine, thus causing that the accumulated path difference causes interference at the end of the structure, which can be further observed through readout of the transmitted spectrum.

A few non-interferometric methods were also reported for the measurements of twist induced linear birefringence change. An interesting approach was reported in [29], where an all-fiber, Distributed Back-Reflector (DBR) laser was created by inscribing FBGs directly into an erbium doped fiber to define the DBR laser cavity. The distance between FBGs was short (10 mm), so that the laser oscillated with a single longitudinal mode. Er-doped fiber in-between FBGs thus defined an all-fiber laser cavity, which was exposed to measured torsion/rotation. The torsional twist imposed to the Er-doped fiber (which possesses intrinsic birefringence, typically at the order of 10^−7^) caused modulation of this intrinsic birefringence and, consequently, the formation of two very similar, but distinctive optical paths (cavity polarization modes) within the laser cavity. This resulted in a splitting of the laser’s output frequency, which was proportional to the twist imposed on the cavity made of the Er-doped fiber. Since the difference in oscillating frequency of both modes was very small, beating of the modes in the radio frequency domain was used to obtain a measurement signal.

## 3. Sensor Based on Modulation of Radial Shear Stress Distribution

**Figure 7:**
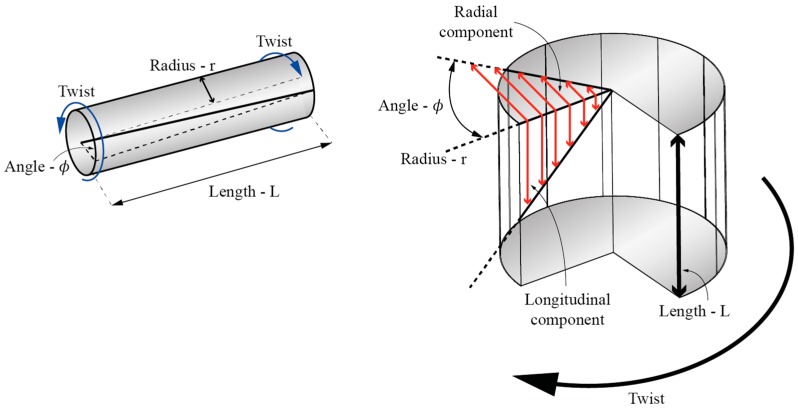
Shear stress distribution along the twisted rod/fiber.

When a solid rod, for example an optical fiber, is exposed to torsional twist around its longitudinal axis, a twist induced share stress is generated with the twisted rod/fiber [30]. The share stress is zero at the center of the rod/fiber and increases linearly proportional towards the rod/fiber’s outer edge, where it reaches the maximum value (Figure 7). The share stress vector can be decomposed further into longitudinal and radial components (the first being parallel with the fiber’s axis and the second perpendicular to the fiber’s axis). Stressed regions of the fiber undergo refractive index changes, which are proportional to local stress and the material’s elasto-optics coefficients. This means that the stress induced refractive index change in a twisted optical fiber will be the highest at the outer edge of the fiber and zero in the center of the fiber.

Fiber modes that are confined close to the center of the fiber, like the fundamental mode, will, thus, see the lowest change in their effective refractive indices, while modes with the fields that extend far out into the cladding will see the highest change in their effective indexes. Thus, phase constant difference among, for example, the fundamental mode (confined to the center of the fiber) and one of the higher order or cladding modes, becomes twist/torsion deepened. Measurement of this difference can, thus, be used for fiber twist/torsion measurement. There are many ways to design twist rotation sensors based on the above described effect, which can be classified roughly in the following groups:

(A) Sensors based on Long Period Fiber Bragg Gratings (LPFBGs).
(B) Sensors based on mode interference effects.
(C) Multicore fiber based sensors.

### 3.1. Sensors Based on Long Period Fiber Bragg Gratings (LPFBGs)

An established method to observe phase constant difference among fundamental and cladding (or in some cases higher order guided) modes is based on applications of LPFBGs. These types of sensors were investigated in great detail over the past decade. When an LPFBG with fixed/known period Λ is introduced to, for example to an SMF, strong coupling will occur between fundamental and cladding modes. This coupling occurs at resonance wavelengths, which are governed by phase matching conditions. Coupling resonance wavelength between the fundamental and particular cladding mode can be described as [31,32]:

(1) $$\lambda_{res}=\left(n_{eff\_fund}-n_{eff\_clad}\right)\Lambda$$

where *n_eff_fund_* and *n_eff_clad_* are fundamental and observed cladding mode effective indexes respectively. Thus, when fiber is exposed to twist/rotation, *n_eff_clad_* changes significantly in comparison to *n_eff_fund_*_,_ which, consequently, leads to change in the resonant wavelength. Since the fundamental (core confined) mode is little dependent on the twist/rotation, the change in resonant wavelength can be approximated as:

(2) $$\Delta \lambda_{res}\approx -\Delta n_{eff\_clad}\Lambda$$

The share stress induced by fiber torsion, which increases linearly in the radial direction of the fiber, has several effects on the properties of the fiber: it modulates local refractive index, it induces circular birefringence and, in the presence of linear birefringence, it modulates the linear birefringence of the fiber. Modulation of the refractive index affects the resonance conditions and, thus, causes shift in resonance wavelengths, while the inducement of circular birefringence can lead to removal of (circular) mode degeneracy, which might lead to different solutions for both circular polarizations that can cause broadening or splitting of the resonance peak into two sub-peaks. This splitting shall be proportional to the torsion applied to the fiber. Modulation of linear birefringence (if present) shall have similar effects.

Since the grating inscriptions’ techniques have strong impact on the appearance of linear birefringence, the (spectral) response to the fiber twisting depends on the selection of LPFBG inscription technique and grating configuration. To design and manufacture an LPFBG torsion sensor, in principle, any known LPFBG inscription method can be used. These include methods of LPFBG fabrication with ultraviolet (UV)-light [33,34], electric arc induced perturbations [35], mechanical corrugation [36], mechanical load (microbending) [37,38,39,40] and application of CO_2_ laser induced perturbation [41,42,43,44,45,46,47,48,49,50,51,52,53].

The combined effects caused by net effective refractive index change of the higher order modes, appearance of circular birefringence and modulation of the degree of pre-existing linear birefringence (also induced by grating inscription) can give rise to complex LPFBGs’ spectrum evolutions, which lead to different possible sensor configurations with different measurement response as described further below.

#### 3.1.1. Torsion Sensors Based on Mechanically Induced LPFBGs

**Figure 8:**
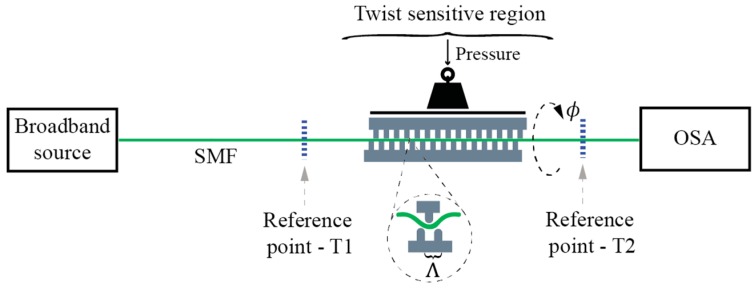
Mechanically induced LPFBG (M-LPFBG) twist/torsion test setup.

Mechanically induced LPFBGs (MLPFBGs) have attracted considerable interest in the past. When using this approach, the sensing fiber is squeezed in-between two corrugated plates to impose micro-bending onto the fiber [54,55]. MLPFBG twist/torsion based sensors are simple, flexible in their fabrication, and have good potential for cost-efficient design (Figure 8). They are also relatively easy to study/investigate. Mechanically induced/fabricated MLPFBG’s usually possess a fair amount of linear birefringence, which is induced by the corrugated plates. In some cases, this can lead to the sufficient degeneracy of modes to obtain two linearly polarized solutions. Thus, coupling to the cladding modes in the MLPFBGs can become polarization dependent as, for example, demonstrated in [56] by Polarization Dependent Loss (PDL) and Differential Group Delay (DGD) measurements. Twist/rotation sensors using an MLPFBG sensor can also be built by using a variety of different fibers as, for example, step-index SM fibers [37], dispersion shifted fibers [57], photonic crystal fibers [58] and doped SM fibers [40]. The use of different fiber types requires proper tailoring of micro-bending deformer and mechanical setup of each fiber type.

Twist sensitivity of a standard single mode fiber in an MLPBFG was studied in [37], where the authors examined in detail twist sensitivity of cladding mode resonances and their cross-sensitivity to strain and temperature. The results show how an optimal selection of stable cladding-mode resonances provides a high sensitivity to twist, low sensitivity to the axial strain variation and low sensitivity to temperature variation.

Another example of MLPFBG based twist sensors exploits a Photonic Crystal Fiber (PCF) [39], where twist imposed on the fiber’s grating generates the splitting of the rejection bands into two sub-bands that move further apart symmetrically as the twist is increased. This splitting does not depend on the twist direction, mechanical pressure and modifications of the grating period. The presented configuration also shows low temperature sensitivity under twist. This splitting can be explained by splitting of the higher order mode into two distinctive circular polarized modes (i.e., removal of polarization degeneracy) due to the appearance of twist induced circular birefringence, i.e., in a twisted fiber two resonances’ solutions exist between the fundamental and higher order modes, each for one circular polarization state. Another MLPFBG PCF based fiber torsion-sensitivity was demonstrated in [58]. An alternative concept for improving sensitivity to twist/torsion in comparison to standard single mode fiber, is fabrication of MLPFBG with rear-earth doped fibers [40]. Reported peak wavelength shifts of the rejection bands were up to 25 nm in this work.

**Figure 9:**
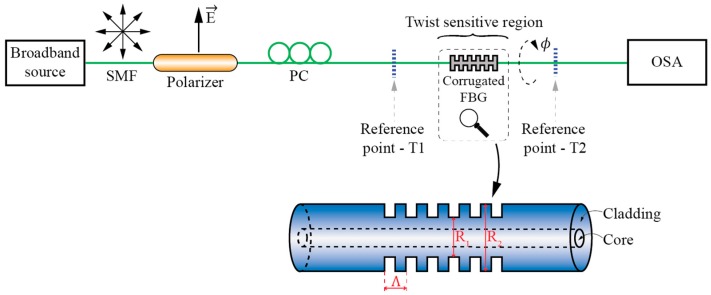
Corrugated LPFBG twist/torsion test setup.

A special type of mechanically induced LPFBG is a corrugated LPFBG, where the cladding of the fiber is etched periodically to achieve the longitudinal perturbation required to induce mode-coupling [57]. Examples of corrugated twist/torsion sensors were presented in [36,59], where the authors described a corrugated structure, made by etching of the fiber (Figure 9). The main mechanisms accounting for the twist sensitive mode coupling are periodic index perturbations. When the corrugated structure is twisted, twist rates differ in the etched (*R*_1_) and not etched (*R*_2_) regions due to differences in the corresponding cladding radiuses. When the fiber is exposed to torsion, this leads to a significant stress concentration in the etched (reduced cladding) areas of the fiber, which causes significant periodic changes in the cladding index modulation, thus shifting the resonance wavelengths to the shorter side of the spectrum, regardless of the direction of the applied torsion.

#### 3.1.2. Torsion Sensors Based on UV Light Inscribed LPFBGs

LPFBGs fabricated with ultraviolet (UV) irradiation [33,34] experience refractive index modulation of the core and far less of the cladding. This is consequence of Ge doping of the fiber’s core and associated core’s photosensitivity that arises from UV induced and Ge-related color centers. Since the index modulation is predominantly confined to a small core area, core mode couples predominantly to the circularly symmetric cladding modes. The dominant twist related measurement effects in these gratings relate directly to the higher order mode effective index modulation, caused by the induced share stress and the appearance of circular birefringence, which are both proportional to the twist applied to the fiber. In comparison to CO_2_ inscribed and corrugated LPFBG-based twist/rotation sensors, variations of resonances wavelengths due to the twist are smaller, but the mechanical strength of the fiber is larger, as there are no structural or mechanical changes imposed on the fiber that could reduce its mechanical strength. Typical reported wavelength shift is 0.05 nm/(rad/m) [34]. Another example of a UV-LPFBG twist sensor which utilizes Polarization Dependent Loss (PDL) measurement, was presented in [33]. In the latter case, the transmission spectra of the UV-LPFBG were different for the polarized light along the two orthogonal birefringent axes, which appeared due to UV-induced refractive index changes between the incident side and the far side of the core. Changes in the PDL peak values encoded information on the fiber’s twist. When there was no twist applied, the PDL spectra had nearly equal peaks, centered around the slow and fast axis resonant wavelengths. After applying twist, peaks’ amplitudes became modulated proportionally to the induced twist rate. Low reported temperature sensitivity was due to equal temperature effect on both slow and fast polarization modes.

#### 3.1.3. Torsion Sensors Based on Electric CO_2_ Laser Induced LPFBGs

**Figure 10:**
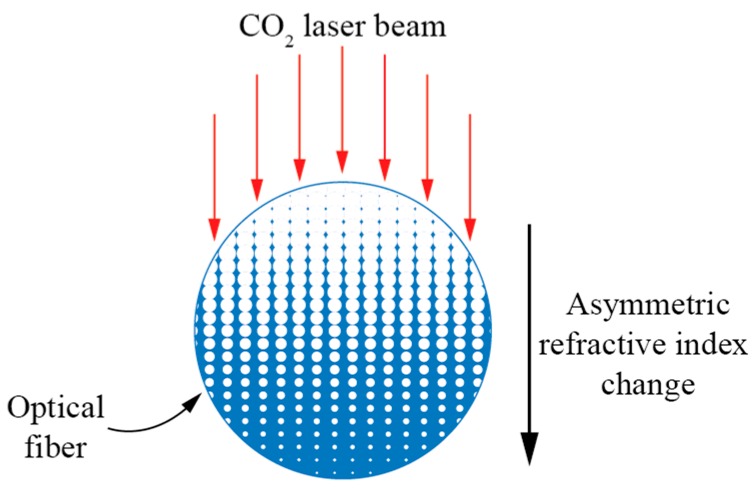
Schematic diagram of asymmetric index profile within a cross-section of a CO_2_-laser-induced LPFG.

LPFBGs fabricated with high-frequency CO_2_ pulses [41,42,43,44,45,46,47,48,49,50,51,52] induced a fair amount of linear birefringence due to asymmetrical distribution of the refractive index change on the cross-section of the LPFBG (Figure 10) which, in combination with the appearance of circular birefringence, gave rise to the manifestation of elliptical birefringence.

Twist induced elliptical birefringence is proportional to the twist rate, while the direction of the elliptical birefringence vector is determined by the twist direction, which causes resonant wavelengths to shifts dependent on the direction of the rotation [41,42,43,45,48,52]. Performance of such a fabricated sensor provides sensitivity in the range of 0.06 nm/(rad/m) [42] with good repeatability and low temperature and strain sensitivity.

**Figure 11:**
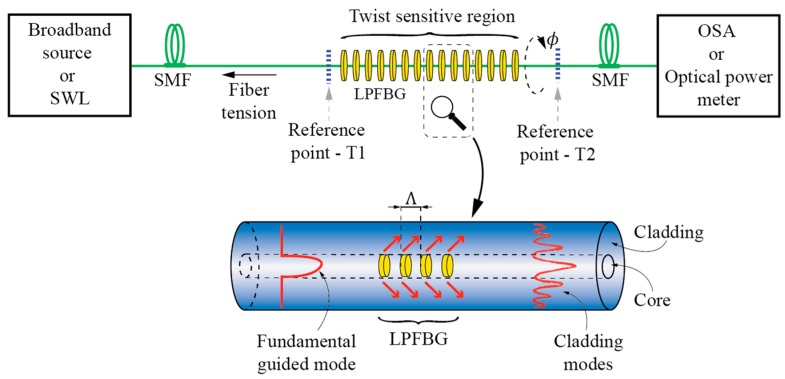
Typical LPFBG twist/torsion test setup.

A highly sensitive twist sensor based on a CO_2_ inscribed LPFBG was presented in [47]. In this work, LPFBGs were inscribed into a Few Mode Fiber (FMF) with a core diameter of 19 µm. Sensitivity of 0.52 nm/(rad/m) within the twist rate range ±100 rad/m was reported with good linearity. Such high sensitivity, when compared with standard SMF LPFBGs, can be attributed to a large (doped) core diameter, which caused strong asymmetrical index modulation within the fiber core, thus provoking a higher linear birefringence and mode coupling between *LP*_01_ and *LP*_11_ (Figure 11). Fabrication of Ultra-Long-Period FBGs (ULPFBGs) also proved to increase twist sensitivity in comparison to standard SMF LPFBGs [51]. The authors in [51] reported fabricating the ULPFBG with a period of 2 mm, which escalated cladding refractive index changes and, thus, increased spectral sensitivity to 0.22 nm/(rad/m) (about four times higher than that of a conventional SMF LPFBG).

Recently, fibers with significant intrinsically induced circular birefringence were proposed to enhance LPFBG sensitivity and to overcome the cross-influence among measured parameters, such as refractive index, strain, torsion and temperature. Two types of these fibers were proposed: LPFBGs with rotary refractive index modulation (R-LPFBG) and fibers with helical refractive index modulation (H-LPFBG).

LPFBGs with rotary refractive index modulation (R-LPFBG) were obtained by exposing the fiber to a high twist rate, while illuminating the fiber periodically by a CO_2_ laser in narrow (100 µm wide) strips/bands to create rotary-frozen-in torsion stress along the fiber. After the grating was relaxed, resonance wavelengths split and this splitting became dependent on subsequently imposed twist rates. Rotary LPFBG sensor setups provide relatively high twist-sensitivity (0.084 nm/(rad/m), while this splitting effect is polarization insensitive while having a temperature coefficient of about 0.07 nm/°C [44]. Recently, fabrication of R-LPFBGs was also reported by use of a femtosecond laser [60], with similarly high twist sensitivity (0.177 nm/(rad/m)).

Another approach in the manufacturing of CO_2_ written LPFBGs with rotary refractive index modulation are Helical Long Period Fiber Gratings (H-LPFBG). In this case, a screw-type periodical index-modulation exists along the fiber axis [34,48,61,62]. For common LPFBGs without any applied twist, the phase-matching condition is described with Equation (1). Due to the helical structure of the H-LPFBG, the twist period is affected (reduced or enlarged) by clockwise or counter-clockwise externally applied torsion. Thus, the change in period ΔΛ becomes a dominant factor to affect the shift of the resonance wavelengths, which can be expressed by [46]:

(3) $$\Delta \lambda_{res}=\left(n_{eff\_fund}-n_{eff\_clad}\right)\Delta \Lambda -\Delta n_{eff\_clad}\Lambda$$

Furthermore, when torsion is applied to the helical fiber, *ΔΛ* and *Δn_eff_clad_* are in an inversely proportional relationship, which enhances resonance wavelength shifts in the H-LPFBGs further. Reference [46] presented a sensor employing HLPFBG written in a two-mode fiber. The achieved twist sensitivity of 0.47 nm/(rad/m) was well above the sensitivity reported in a comparable sensor utilizing a conventional CO_2_ laser written LPFBG. The temperature sensitivity (23.9 pm/°C) was also lower in comparison to conventional LPFBGs.

Further work in this area led to Multi-Phase Shifted Helical LPFBG (MPSH-LPFBG) sensors [49] that employ a multi-period rotation technique with π/2 and 3π/2 phase shifts in the MPSH-LPFBG period, thus creating three loss-dips in a selected loss band. The increase of the rotation angle shifts these peaks simultaneously towards longer wavelengths, however, with different rates. Thus, interrogation of wavelength differences between individual peaks allows for measurements of twist rate. With the increase of the temperature, peaks also shift to a longer wavelength, but, in this case, the wavelength distance between the two-phase shift peaks remains almost unchanged, thus minimizing temperature cross-sensitivity. Another twist sensor incorporated a pair of H-LPFBGs with opposite helicities which, in the proposed setup, also facilitated temperature measurements was presented in [61].

#### 3.1.4. Torsion Sensors Based on Electric Arc Induced LPFBGs

The process of LPFBG inscription by electric arc discharges is related closely to other techniques, such as CO_2_ laser radiation, which employ local heating of the fiber. They can be written in any type of optical fiber, such as standard telecommunication fibers [63], PCF fibers, holey fibers and doped fibers [64,65]. The concern in arc gratings is Polarization Dependent Loss (PDL), which results from birefringence induced in the fiber core due to one side exposure and these values depend on fiber fabrication parameter, such as pulling tension [66]. Another implementation described in [35] represents a self-referenced twist sensor with simple demodulation technique. This approach eliminated the need for a spectrum analyzer and relied on a Mach-Zehnder configuration that utilizes an external independent reference fiber arm. Modulation in the radiofrequency domain was used to obtain self-referenced frequency response proportional to the measured signal.

### 3.2. Sensors Based on the Mode Interference Effects

**Figure 12:**
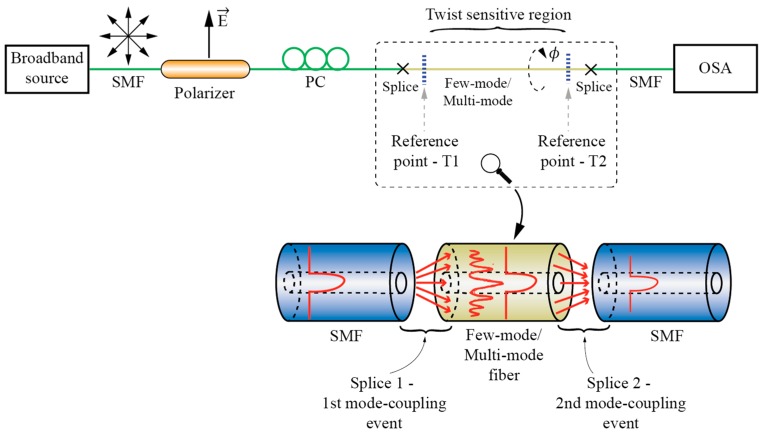
Fiber Mach-Zehnder interferometer for twist/rotation sensing.

When two or more modes with different field confinements and, consequently, different effective index dependences on the fiber twist rate, are coupled locally at two spatially dislocated points along the sensing fiber, an in-fiber Mach-Zehnder interferometer is formed that is sensitive to the fiber’s twist/torsion [67,68,69,70,71,72,73,74]. A basic example of modal interferometer for twist/torsion sensing is shown in Figure 12, which consists of two spatially-dislocated mode-coupling events that couple two (or more) modes with different field confinements.

Mode coupling events can be realized in various ways, but the approach used most frequently is to use splices in-between dissimilar fibers [67,68,69,70,71]. Other methods are also possible, like core offsets [72], abrupt tapers [75], LPFBG or similar. Michelson interferometer configuration is also possible by replacing one coupling event by a mirror [68], which allows for design of a compact sensor system that utilizes a single lead-in fiber.

**Figure 13:**
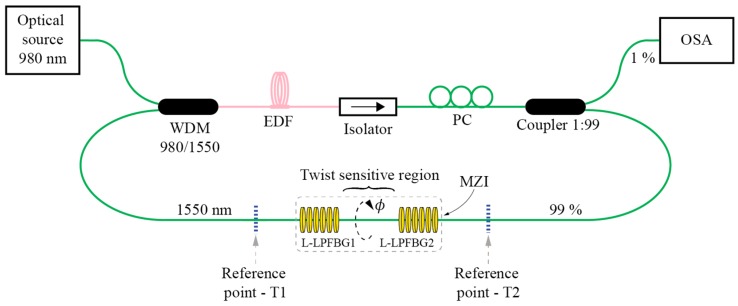
Torsion sensor employing Mach-Zehnder LPFBG in fiber ring laser configuration.

Modal interferometers can utilize coupling among guided modes [67], guided and cladding modes [68,70,71,72] or a combination of both approaches [69]. While solid core fibers can be used to build torsion sensitive fiber modal interferometers [72], most solutions utilize PCF, mainly to achieve low temperature sensitivity [67,68,69,70,71]. A rather special case of modal interferometer is the solution presented in [73], where a large square core fiber is applied as a multimode interferometer for twist/rotation sensing. Another case of twist modal interferometer was presented in [73], where a twisted taper pair is produced within polarization maintaining fiber to provoke core-cladding torsion sensitive interference. Fiber Mach-Zehnder Interferometers (MZI) can also be built by using LFBGs. One example of a torsion sensor was demonstrated in [50], where two cascaded R-LPFBGs formed a torsion-sensitive region, which was demodulated with a fiber ring laser. This solution provides a narrow laser linewidth with high side-mode suppression ratio, which increased the torsion measurement resolution significantly (Figure 13). A similar approach was described in [76], where the authors formed a cascaded LPFBG in Polarization-Maintaining (PM) fibers. An additional example of MZI was also presented in [77], where the authors cascaded two identical Helical Long Period Fiber Gratings (H-LPFBGs).

Another, rather more special example of a core-cladding interference twist/torsion sensor was presented in [78], where a continuously twisted solid-core PCF was used as a sensing element (the fiber was pre-twisted permanently by localized heating using a CO_2_ laser). Continuously twisted solid-core PCF supports angular momentum states in the micro-structured cladding and these states couple to the core mode, giving rise to a series of resonance dips in the transmission spectrum, which respond to fiber twist/rotation, due to the modulation of the cladding states indexes.

### 3.3. Multicore Fiber Based Sensors

**Figure 14:**
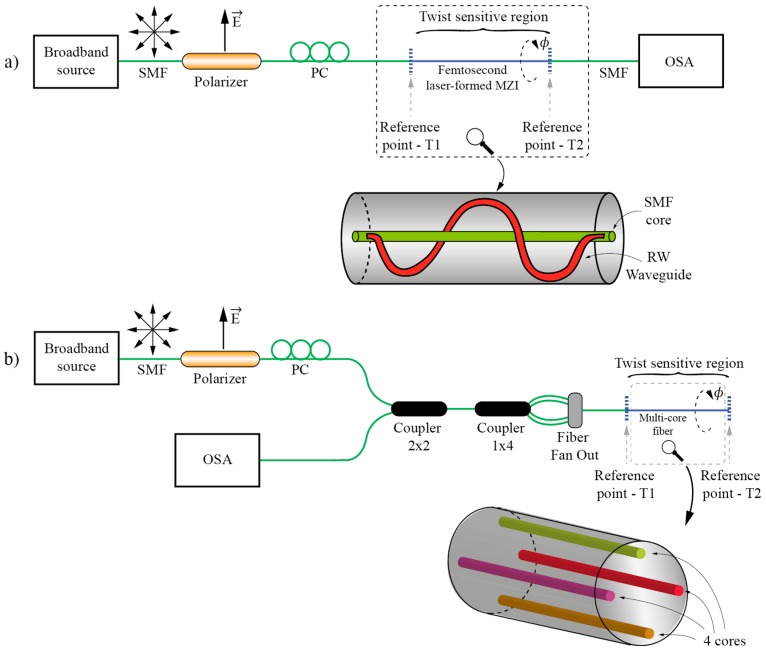
(**a**) Helical twist/rotation sensor setup; and (**b**) Multicore twist/rotation sensor setup.

A relatively straightforward way to detect variations in the share stress distribution caused by twist/torsion in the transversal plane of the fiber is to use multicore fibers. When two (or multiple) cores are present at different radial positions within the fiber cross-section, shear stress will modify their indexes differently. The most straightforward way in the design of multicore twist/rotation sensors is to implement one core in the center of the fiber and one core at the fiber’s periphery. The sensitivity of such a sensor may be increased further by spinning the fiber during drawing, which creates a helical multicore fiber. Inscriptions of FBGs into central and peripherally positioned helical cores, while subtracting their characteristics’ wavelengths, allow for unambiguous (temperature and strain compensated) read-out of the fiber’s twist/torsion [79,80] (Figure 14a).

An interesting approach in the design of a helical multicore sensor was reported in [81], where helical cores were written into a standard SMF by the use of a femtosecond laser. Two approaches were demonstrated: a version where an in-fiber Mach-Zehnder was formed by coupling an SMF core with a laser-written helical core, and a version where two helical cores with inscribed Bragg gratings and with opposite helices’ rotation direction were coupled to the SMF core, which allowed for easy access to both gratings through the same SMF core. This sensor allowed for compensation of axials’ strain and temperature (Figure 14b).

When using PCF fibers, the cores can also be very small and very close to one another. This allows for simple creation of in-fiber Mach-Zehnder interferometers by splicing a bigger (SMF) core over such closely spaced cores of dual/multicore core PCFs. Minute variations in both cores’ circular symmetry are then sufficient to induce variation of optical path length in both cores due to the induced fiber twist/rotation [82]. A similar approach was also present in [83].

## 4. Twist/Rotation Sensors Based on the Spatial E-Field Displacement

**Figure 15:**
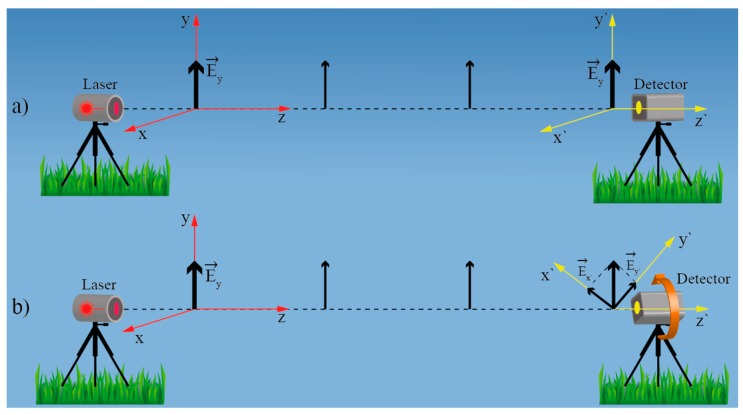
(**a**) E-field vector orientation remains unchanged during propagation through isotropic medium; (**b**) Rotation of the receiver rotates E-field vector in receiver’s local coordinate system.

Arguably, this is the most basic, and perhaps the most straightforward, approach to the twist/rotation sensing that utilizes electromagnetic radiation. When a linearly polarized electromagnetic wave is generated in a reference frame which is filled with homogenous and isotropic medium, the wave’s E-field vector orientation in the reference frame will remain unchanged over the entire path of the wave’s propagation (homogenous isotropic medium cannot change the polarization state of a propagating electromagnetic wave (Figure 15a). Thus, if a linearly polarized source and polarization sensitive receiver are placed collinearly at an arbitrary distance, rotation of the receiver relative to the source will cause rotation of the E-field impeding at the receiver. Thus, measurement of E-field vector orientation direction at the receiver provides direct information on rotational displacement between the transmitter and receiver (Figure 15b).

**Figure 16:**
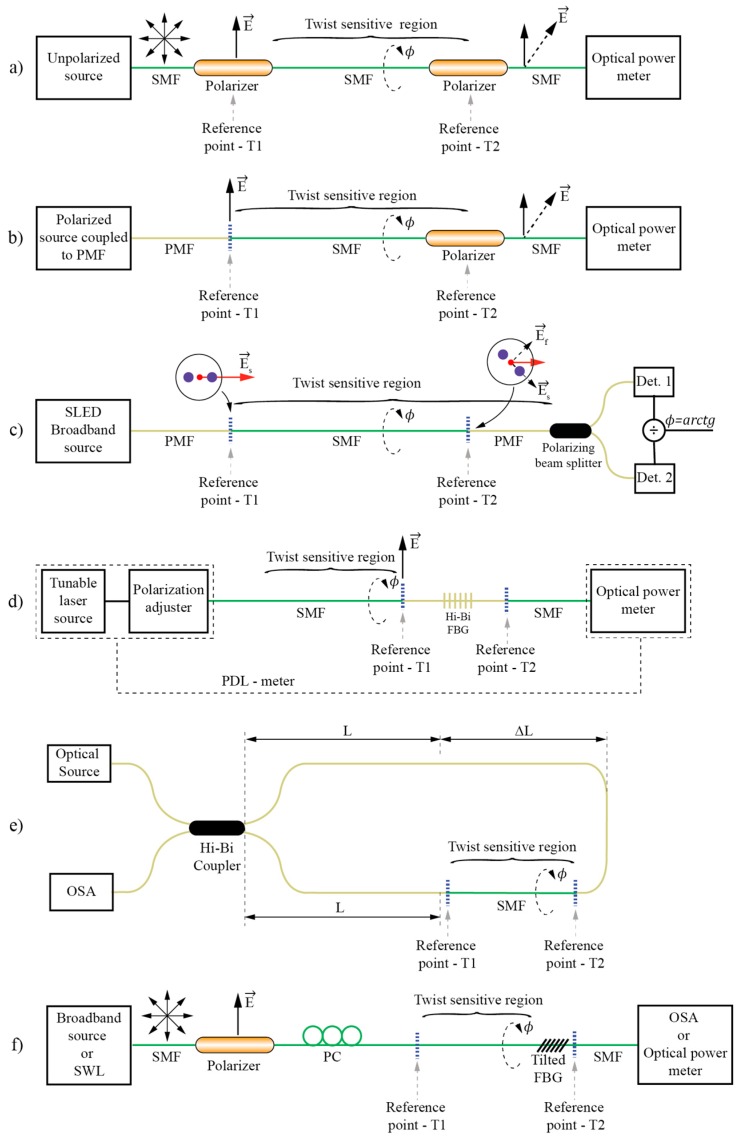
Typical E-field vector displacement rotation sensors’ arrangements.

Ideal, perfectly circular, linear birefringence free, and straight single-mode fiber is a one-dimensional equivalent of a homogenous and isotropic medium, except that it can confine and guide light from the transmitter to the receiver effectively. Ideal, circularly-symmetric and straight fiber cannot rotate or otherwise affect the polarization plane. For any polarization transformations to take place within the fiber, the circular symmetry must be broken, which results in the appearance of (weak or strong) birefringence(s). Short sections of straight, modern telecom single-mode fiber, like SMF-28, are actually a very good approximation of ideal circularly-symmetric fiber. A significant effort has been devoted to fiber production process optimization to remove any intrinsic birefringence from these fibers due to the polarization mode dispersion effects that otherwise affect telecom systems adversely. Thus, by placing a linearly polarized source at one side of a short and straight section of SMF, and a polarization analyzer on the other, rotational displacement between source and polarization analyzer can be determined by measuring E-field vector orientation incident at the polarization analyzer as shown, for example, in Figure 16a.

Twisting of straight fiber does not break circular symmetry and, thus, does not cause any polarization state transformation. Twisting, however, does cause the appearance of a weak circular birefringence (as already discussed), which is reflected in a rotation of the polarization plane along the fiber. This additional rotation is, however, relatively small in comparison to the twist imposed to the fiber and which causes rotation through opto-elastic effect. For example, twisting of SMF by 1 degree causes rotation of the polarization plane within the fiber by about 0.069° [84]. This polarization plane rotation is the opposite of the mechanical rotation of the fiber, thus, the rotation angle measured by an analyzer at the fiber output must be multiplied by a factor of 1.069 [84] to obtain the correct twist angle readout. The appearance of twist induced circular birefringence (optical activity) is, thus, not limiting in measurements of rotation angles by using the E-field vector displacement measurement principle. The only adverse effect that comes from the appearance of circular birefringence is, perhaps, in a slight increase of the system’s temperature sensitivity. While the twist induced circular birefringence is temperature sensitive, its contribution to the angle readout is small, and will, thus, cause only very limited temperature dependence (for example when the fiber is twisted through 90°, a 50 K temperature change of the same fiber will induce error in angle readout of less than 0.4° [84]).

There are, however, certain practical limitations that need to be considered when utilizing circularly symmetric fibers in E-field vector displacement sensor setups: (a) firstly, the fiber must be sufficiently straight. Fiber bending gives rise to the appearance of weak linear birefringence, which can cause polarization state transformation along the fiber. Thus, the amount of bend-induced linear birefringence must remain sufficiently low to prevent any measurable adverse effects. While this limitation was not treated systematically in the available literature, tests conducted in [85] show that, when the sensing fiber section is a few meters long, the bend radius above 2 m does not induce any errors that would exceed 0.2-degree readout errors in a final measurement setup. It is also shown in the same reference that localized microbeads are also relatively benign and tolerable as long as perturbation is not severe (normal contacts of fiber with packaging elements, like housing, or proper cabling do not present a concern); (b) Secondly, there is a question of how long the SMF section can be while still preserving sufficient quality of initial polarization? While this depends strongly on the quality of the fiber (intrinsic fiber birefringence shall determine this limit), sensors with sub-degree resolution were demonstrated using a 50 m long section of Corning’s SMF-28e. It shall be stressed that most other principles (like sensors based on LPFBG or modal interferometers), also require straight fibers and were never annualized in configurations that measure rotation over longer distances.

The approach described above allows for design of a variety of sensors’ configurations. Figure 16 shows typical possible approaches. For clarity, Figure 16 denotes fixed and rotary reference points, denoted by T1 and T2 respectively. The presented sensors measure rotation between T1 and T2 and around the axis that passes through T1 and T2. T1 and T2 are always interconnected by a straight section of SMF. In Figure 16a a non-polarized fiber source, like ASE, is coupled to the SMF. Two linear polarizers are introduced at fixed (T1) and rotary (T2) reference points. When the axes of both polarizers are aligned, the transmission is maximal, and when the polarizer at T2 is rotated by 90°, the transmission goes to zero. For angles in between, the transmission follows *cos^2^* (Malus) law, i.e., the present setup forms an all-fiber Malus setup, thus forming a simple, intensity based rotation sensor. A broadband non-polarized source can also be replaced by any polarized source (for example laser diode) coupled into one of the polarization maintaining (PM) fiber axes. The PM fiber then connects to the SMF at fixed reference point T1. This approach was used, for example, in [85] for design of a quasi-distributed twist/rotation sensing system as discussed further below. The setup shown in Figure 16c eliminates most of the drawbacks associated with the intensity setups presented in Figure 16a,b as it introduces ratio-metric measurements that compensate automatically for source power and loss fluctuations in the fiber systems [84]. Here, a broadband polarized source (like SLED) is spliced to the PM fiber. The PM fiber is brought to the reference point T1, where it connects to the straight measurement section of SMF. This assures launching of linearly polarized light into the SMF. At rotary reference point T2, the SMF is connected back to the PM fiber. Thus, when the second fiber splice at T2 between the SMF and PMF is rotated relative to the first splice at T1, the incoming linearly polarized E-field vector excites both modes of the output PM fiber. The PM fiber is then connected to a polarization splitter that directs light from the fast and the slow axis of the PMF fiber to two different detectors. Any further rotation of the PM fiber does not have any effect on the mode power ratio as high birefringence in the fiber prevents power exchange among the modes as already described above. Finally, powers measured at both detectors are divided and the square root of this ratio presents the *ArcTan* of rotary displacement between T1 and T2 [84]. In this setup, the coherence of the source must be sufficiently low and/or the PM fiber sufficiently long to prevent modal interference within the output PM fiber (when using typical SLED a few meters of PM fiber is sufficient to comply with this condition). Change of incident polarization at the output of an SMF can be also encoded spectrally if so desired. Figure 16d,f shows such examples. Figure 16d shows an example described in detail in [86] where a tunable laser source and a polarization adjuster are utilized to launch light into the SMF at reference point T1. At rotary reference point T2, there is a short section of HB fiber with inscribed FBG. When FBG is inscribed in an HB fiber, two characteristics’ FBG wavelengths are obtained, each belonging to one of the HB fiber’s polarization modes. Thus, if polarization at the HB fiber’s input is changing (due to the E-field rotation) the spectral transmission characteristic will also change accordingly (when input E-field vector is aligned with the slow axis, loss will be maximal at shorter FBG resonance and, when the E-field vector is aligned with fast axis, the loss will be maximal at longer FBG resonance). In the reference [86] the authors configured their system for PDL measures and they deduced the rotation angle from the ratio of PDL loss peaks’ ratios; however, the system could also be simplified considerably by omitting the polarization adjuster and providing, for example, linearly polarized broadband light at the SMF input while simply observing loss dips at both FBGs’ resonances.

Another interesting example of spectral encoding was described in [87] and shown in Figure 16e, where SMF exposed to the twist/rotation was integrated into a Sagnac interferometer consisting otherwise from HB fiber. The SMF was placed asymmetrically into the loop to form an unbalanced Sagnac loop; the SMF exposed to rotation then acted as a rotation dependent polarization mode exchanger, leading to the interference of four possible waves having four possible paths, i.e., light components exiting from each fiber’s polarization axis couple back to another two axes, while this coupling depends on the imposed rotation. This leads to the formation of a double interferometer and fringes of this double interferometer beat to form a more complex spectral pattern, which encodes the twist/rotation present along the SMF fiber.

Figure 16d shows a system that uses a broadband source, lead-in fiber and linear polarizer at reference point T1, which launches linearly polarized light into the measurement section of SMF that interconnects T1 and T2 in the same/similar way as in the previous examples. At the rotary reference point T2, a tilted FBG is inserted, which couples a fraction of the light to the cladding modes in a resonance way. Since the tilted FBG breaks down the circularly symmetry of the fiber strongly, this resonance coupling depends on the state of polarization at the input of the tilted FBG, thus, the tilted FBG encodes polarization vector orientation into the specific signature in the transmitted (or reflected) spectrum. The latter is analyzed further by an Optical Spectrum Analyzer (OSA) to decode the in E-field vector orientation and, thus, the rotation angle between T1 and T2. More details on the tilted FBG system will be discussed in detail further below as these types of sensor have attracted a substantial interest and are thus discussed separately.

There are also other methods that employ spectral encoding of E-field orientation rotation. An interesting example was presented in [88], where a small fiber loop at the end of the twisted SMF was configured as a polarization sensitive Mach-Zehnder interferometer, which encoded incoming E-field orientation spectrally. Another example of spectrally encoded E-field displacement rotation sensor was presented in [89], where a combination of Malus arrangement (similar to Figure 16a) and Fabry-Perot cavity were used to demonstrate rotation measurements. This system, however, utilized a bulk-optics arrangement.

**Figure 17:**
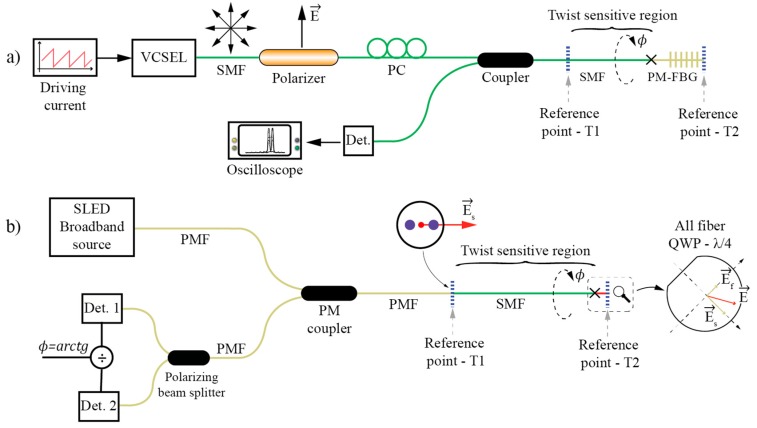
E-field vector displacement rotation sensors using single lead fiber solutions with E-field rotation encoding employing (**a**) An FBG inscribed in the Hi-Bi fiber; and (**b**) An all-fiber wave-plate.

All the solutions described above are two-port solutions, meaning that the SMF defining bend sensitive region must be accessed from both sides, i.e., using lead-in and lead-out fibers. This might be quite an important limitation in practical sensor design, as already discussed in previous sections. While the total elimination of the need for accessing the SMF on both sides is not viable, one side can be replaced by a compact and all-fiber polarization encoder. Two such designs were reported in the literature so far, shown in Figure 17. In [90] the authors used a polarized tunable laser source (low-cost design was archived by current tuning of VCSEL), which excited the sensing section of an SMF through an optical coupler. The other (far) side of the sensing SMF was spliced to a short section of HB fiber with inscribed FBG. Due to the intrinsic birefringence in the HB fiber, two resonance FBG wavelengths existed and reflectance from the FBGs deepened on the incident light wavelength and E-field orientation relative to the HB fiber principal axis. Thus, rotation of the E-field vector at the HB-fiber input causes variation in reflectance amplitudes at characteristics FBG wavelengths, while the ratio of those reflections encodes SMF twist/rotation (Figure 17a).The system is similar to the one described in Figure 16d and reference [86], except that it provides a more practical and cost efficient arrangement. Another single-lead-fiber version was described in [91]. Here, the setup described in [84] and Figure 16c is modified to allow access using a single fiber. To achieve this, linearly polarized light was again launched into the sensing section of the SMF, while using a quarter wave-plate and mirror at the end of the sensing SMF fiber. The quarter wave-plate used in reflection mode (with a mirror at the end) acts as half wave-plate, which flipped the polarization plane by 90° and, thus, allowed for E-field rotation encoding in a back-reflection mode (if a simple mirror would be used at the end of the SMF one could not detect rotation, as the E-field vector in the reference frame would remain unchanged). When using all-fiber wave-plates (produced out of HB fibers) there is a challenge to design a system that is not temperature sensitive. This was achieved in [91] by a combination of different HB fibers (Figure 17b).

**Figure 18:**
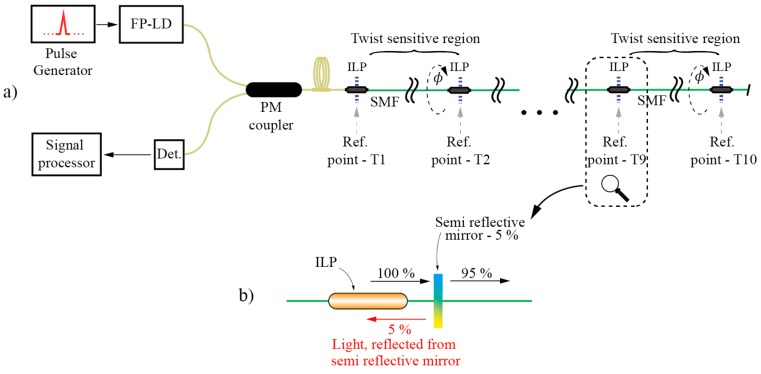
(**a**) A quasi-distributed sensor array for twist/rotation setup; and (**b**) ILP with a semi-reflective mirror setup.

As already mentioned, E-field vector displacement rotation sensors can be multiplexed to form a quasi-distributed rotation sensor array [85]. This is a unique property of the E-field displacement approach. In [85], special in-line fiber polarizers with high return loss were built, i.e., semi-reflection mirrors were placed behind polarizers, as shown in Figure 18b. Then, an array of these polarizers was interconnected with a straight SMF section (Figure 18a). The system was interrogated with very simple OTDR (consisting of an ordinary Telecom laser diode and PIN detector—mirrors provided high reflectance, thus, there was no need for a more complex detection stage). A single launched pulse produced a train of back-reflected pulses and ratios among neighboring pulses’ amplitudes encoded twist/rotation imposed to the individual sensor segments. This setup can be viewed as a sequence/chain of multiple all-fiber Malus arrangements as shown in Figure 16a (with added mirrors for easier signal interrogation).

**Figure 19:**
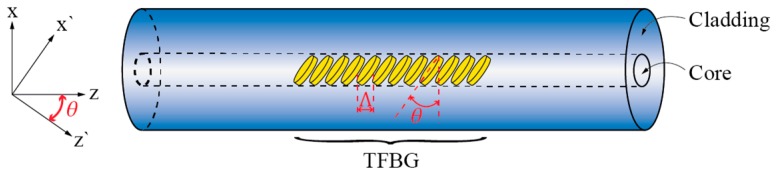
Tilted FBG formation.

Torsion sensors based on Tilted Fiber Bragg Gratings (TFBG) are another group of sensors that exploit E-field displacement in circularly symmetric fibers as already mentioned above. Here, we provide a bit more detailed introduction as these sensors have gained quite some attention in the research community. TFBG is a fiber device produced by inscribing an FBG in a direction other than normal to the axis of the fiber as shown in Figure 19.

**Figure 20:**
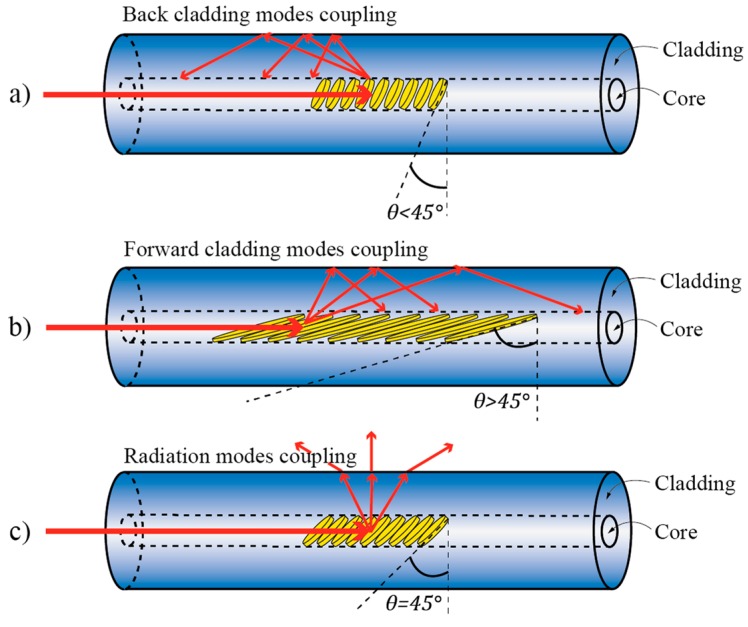
Feasible arrangements for forward and backward coupling of cladding modes in TFBGs. (**a**) Back cladding modes coupling; (**b**) Forward cladding modes coupling; (**c**) Radiation modes coupling.

In general, this type of grating can couple light from the fundamental core mode to the cladding modes of the fiber effectively. A detailed theatrical background on the coupling process and conditions in TFBGs is, for example, presented in [92]. This coupling occurs in a resonant way, i.e., when phase matching conditions between the guided core and the cladding modes are met [93,94]:

(4) $$\lambda_{FM-CLM}=\frac{\Lambda }{\cos\theta }\left(n_{eff\_FM}\pm n_{eff\_CLM}\right)$$

where *n_eff_FM_* is the effective index of the fundamental core mode, *n_eff_CLM_* the effective index of the *m*-th cladding mode, *θ* is the grating tilt (measured as the angle between the grating tilt plane and plane normal to the fiber axis), and *λ_FM-CLM_* represents the resonance wavelength at which mode coupling occurs. Depending on the grating tilt *θ*, the mode coupling may be divided into three regimes [92] as shown in Figure 20: if the grating tilt is less than 45°, the forward propagating mode will be coupled to the backward propagating cladding (or radiation) modes (Figure 20a). If the grating tilt is more than 45°, the forward propagating mode will be coupled to the forward propagating cladding (or radiation) modes (Figure 20b). If the tilt corresponds *θ* = 45°, then the light can be coupled out of the fiber and this condition can be used to build fiber polarizers (Figure 20c) [95]. Forward-propagating configurations (grating tilt is more than 45°) are, perhaps, used most frequently in twist/rotation sensing, as shown further below, due to the very distinctive polarization response.

Similarly, when a TFBG is written into a multimode fiber, resonant coupling among guided or guided and cladding modes can occur [96] in similar ways as described above for the case of single-mode fiber.

**Figure 21:**
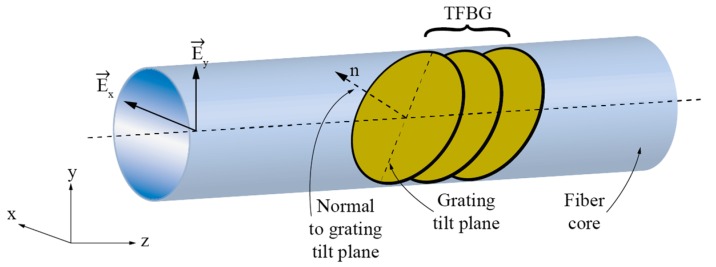
Each polarization state (E_y_ and E_x_) yielding to different resonant solution due to its corresponding orientation.

The principal property that makes TFBGs interesting for twist/rotation measurements belongs to the grating’s ability to break the local circular symmetry of the fiber, which makes resonant coupling between the core and the cladding modes depend on the incoming E-field vector orientation. This leads to two distinctive sets of resonant coupling conditions/wavelengths (each set consists of multiple resonance wavelengths at which coupling occurs between fundamental and multitude of cladding modes): One set for the case when the incident E-field vector is parallel to the grating’s tilt plane, and one set for the case when the E-field vector is parallel with the plane normal to the grating’s tilt plane (Figure 21).

**Figure 22:**
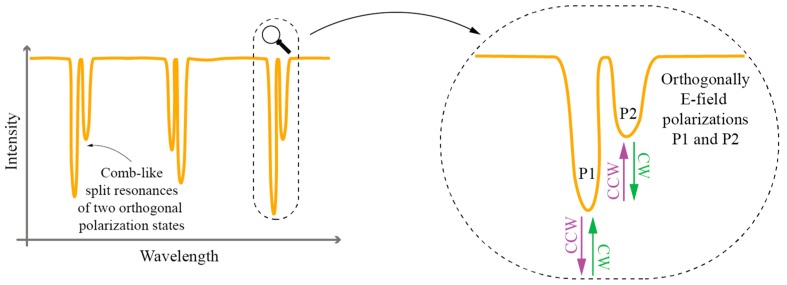
An example of forward propagating spectrum in excessively tilted FBG and its spectral response; ratio of dips P1 and P2 changes with fiber rotation (CW/CCW).

The resonant wavelength at which coupling occurs between fundamental and particular cladding modes thus becomes split into two closely spaced resonances, each belonging to a particular polarization state. This polarization dependence is especially pronounced in excessively tiled gratings [94,97]. Rotation of the incident forward propagating E-field vector around the fiber axis therefore leads to periodic variation in coupling strength at resonant wavelengths as shown in Figure 22. The TFBG spectrum thus encodes information on the incident E-field orientation. For example, observation of coupling strength at a particular resonant wavelength (i.e., depth of an intensity dip in the transmission spectrum), or ratio of coupling strengths (ratio of intensity dips) belonging to two orthogonal E-field polarizations [94,95,97], thus carries information on the incident E-field direction relative to the TFBG tilt plane as, for example, shown in Figure 22.

**Figure 23:**
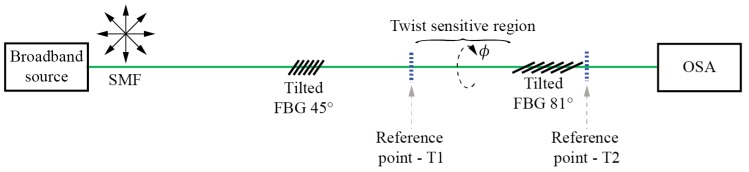
TFBG setup for rotation measurement.

The capability of a TFBG to encode input E-field vector orientation into a distinctive spectral response can be applied straightforwardly in the E-field displacement type of rotation sensors. A common rotation sensor setup that utilizes TFBGs thus consists of a polarized optical source, lead-in fiber, in-line fiber polarizer, a section of sensing (circularly symmetric) SM fiber (usually standard SMF), a TFBG, a spectrum analyzer, and lead-out fiber, as shown in Figure 16f, where the section of the sensing SMF fiber with TFBG at its end are exposed to measured twist/rotation. When the rotational alignment between source/polarizers and TFBG is changed, the E-field vector spatial orientation incident onto the TFBG is also changed, which modulates resonance dips in the transmitted optical spectrum. The polarizer in the system can be either all-fiber, bulk/micro-optic [94] or another 45° TFBG [95] (Figure 23).

While the setup described above and its variations are the most commonly found in literature, other variations using TFBG are also possible. In [98] a TFBG based sensor was placed into a fiber-laser cavity. Twisting of the SMF fiber and TFBG modulates cavity loss, which is reflected further in laser oscillation build-up time variation. Twist/rotation angle was determined by measuring this time.

**Figure 24:**
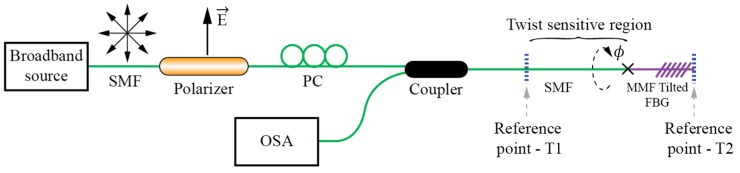
TFBG setup for twist/rotation measurements employing FBG’s inscribed in multi-mode fiber.

Reference [99] describes torsion sensors based on TFBG inscribed into multimode fibers (Figure 24). Multiple high-order core modes can be excited by the TFBG inscribed in an MMF and they show a well-defined “comb” of resonances which are well separated from each other spectrally (this provides an easy way to isolate them at different wavelengths). Because the introduction of tilted grating has the effect of breaking the circular symmetry of the fiber locally, the coupling of asymmetric modes (*LP*_1m_) depends strongly on the polarization orientation related to the tilted plane, while the symmetric modes (*LP*_0m_) do not. In [99], a selective launch of symmetric modes (predominately fundamental modes) was performed by splicing of the (launching) SM fiber to the multimode fiber.

The observed back-reflected spectrum showed two groups of resonance peaks in the back-reflected spectrum: One group of resonances corresponding to coupling of symmetric (polarization insensitive) modes and the other group of resonances corresponding to coupling of asymmetric (polarization sensitive) modes. Rotation of the SMF/TFBG section thus caused intensity variation of some, but not all dips in the spectrum, and ratio among those dips that showed twist related variation, and those that did not were used to obtain temperature independent rotation sensing.

Another, and rather special, case of TFBG twist/torsion sensor was reported in [100], where an SMF section containing 10° TFBG was coated with a 50 nm thick layer of gold. The sensing part was immersed into water, which created conditions for the excitation of surface plasmons through coupling of light from the fundamental mode to the cladding modes of the fiber. The mode coupling that provoked excitation of the surface plasmon, however, occurred only under specific E-field orientation of the input light, which was further observable in the specific shape of the back-reflected-optical spectrum.

## 5. Other Twist/Rotation Sensors

In this section, we summarize twist/rotation methods that cannot be classified into the previous three major groups described above. When the fiber twist-to-linear-birefringence ratio is sufficiently high, twist/torsion can, by itself, act as a perturbation that can provoke mode coupling among polarization modes of the PM fiber, which can be monitored further, for example, by observation of the spectral response of the FBG written in the same fiber [101]. Whilst this effect is not pronounced soundly in PM fibers, it seems it can still provide a sufficiently distinctive spectral response that can be used for twist/rotation sensing. A similar approach was also reported in [102], where a longer FBG was, firstly, written into the PM fiber, and then tapered in the middle of the grating to form two chirped FBGs separated by a thinned/tapered waist region. A structure like this was then exposed to a twist/rotation. The tapered region concentrated twist induced stresses within the thinned fiber region and, also, increased significantly the susceptibility of polarization modes for the mode coupling as tapering reduced the phase constant difference among both polarization modes (the magnitude of a coupling coefficient among two modes depends strongly on the phase constant difference among modes).

A further example of twist/rotation sensor was presented in [103], where an unpolarized broadband source was used to launch light into a PM fiber with inscribed FBG. The launched light back-reflected at two distinctive wavelengths, each having different orthogonal polarizations (an FBG written in a PM fiber provides two reflection peaks for each linearly polarized mode as already discussed). Reflected light was then fed through a standard SMF to another section of HB fiber that was exposed to twisting, while projecting its output towards optical spectrum analyzer through a linear polarizer (the PM fiber was rotated in front of a linear polarizer, which rotated the PM fiber’s axis relative to the linear polarizer and Optical Spectrum Analyzer). The spectrum analyzer was used to determine the rotation by measuring the power ratio at both FBGs’ characteristics’ wavelengths—for better clarity, a simplified version of this system, without spectral encoding, would simply use a piece of singe-axis launched PM fiber that would rotate in front of a polarizer and detector. This approach is also an extrinsic type of sensor, i.e., just behind the sensing region, the light is coupled out of an optical fiber.

Chiral fibers [104] can also be used for twist rotation sensing, although this approach can also be viewed as a special case of R-LPFBG, as already described in the previous section.

**Figure 25:**
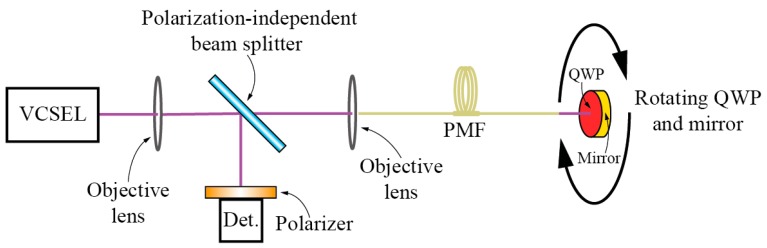
Rotation sensing system using a VCSEL light source with optical feedback.

In [105] an extrinsic fiber optic rotation sensor was presented where a rotatable quarter-wave mirror was positioned in the front of a PM fiber (Figure 25). Relative rotation between the fiber principal axis and the mirror axis was measured by using this fiber-mirror setup as an optical feedback in an active laser system based on a Vertical-Cavity Surface-Emitting Laser (VCSEL). Rotation of the mirror in front of a PM fiber caused the optical powers of two VCSEL polarization modes to change with changing mirror angular position.

**Figure 26:**
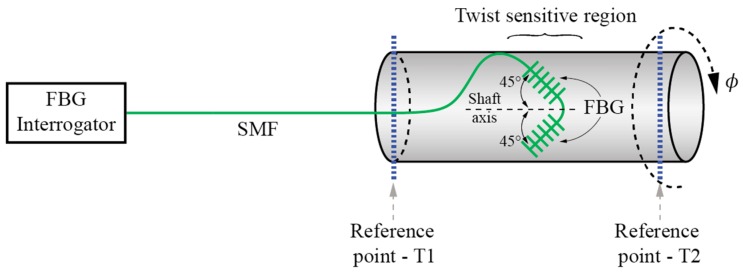
Schematic setup of differential configuration of FBG sensor at 45° angle relative to the shaft axis.

There is also a group of rotation/twist/torsion sensors that utilize conventional approaches for twist/torsion measurements on larger (measurement) bodies like cylinders or solid roads. These approaches have been used for decades to build high-quality torque and torsion sensors using conventional strain gauges. In these configurations, conventional fiber-optic strain sensors (mostly FBGs) are applied to measure directly the shear stress at the surface of the measurement body, while this measured stress is then correlated to twist/rotation or torque. Differential configurations of FBGs or other fiber-optic strain sensors, at a 45° angle relative to the shaft axis (Figure 26) are, thus, commonly employed to enhance the sensitivity and achieve efficient temperature compensation as, for example, shown in [106,107,108,109,110,111]. Similar results can be achieved by specially designed measurements bodies as, for example, reported in [112]. Another interesting approach is a distributed torsion sensor employing cascaded coaxial cable Faby-Perot interferometers mounted on a shaft, where the authors have implemented weak reflectors on a coaxial cable, thus forming between any two consecutive reflectors a Fabry-Perot cavity [113]. Torsion is measured indirectly from the measured axial strain due to applied torsion to the shaft.

Other mechanical setups are also possible [114], for example, describes a system where measured rotation was firstly converted into a linear translation. The latter was transferred further on the surface of a long chirped FBG, which was resolved spectrally to determine input rotation.

Finally, optical fibers (mainly multimode or even POF) can be used to deliver and acquire light from conventional Moire based (rotary) encoders [115]. These arrangements are, nowadays, available commercially [116].

## 6. Conclusions

Fiber based twist/torsion/rotation sensors have been a topic of intense research over the past decade. Different approaches and concepts were proposed and demonstrated. In general, existing solutions can be classed into four main principal categories of sensors: sensors based on birefringence modulation, sensors based on share stress refractive index modulation, sensors based on E-field displacement detection and other approaches. Each of these groups have a distinctive set of properties. Many of these approaches are based on the interrogation of the sensor’s spectral response. While spectrally resolved interrogation techniques are, currently, accepted widely, and preferred in most industrial fiber-optics applications, the relatively high-cost of the spectral interrogator might limit commercial introduction of these approaches in twist/rotation sensing. Twist/rotation sensors are typical single-point sensors with limited ability or demand for multiplexing, and usually cannot tolerate a too high system cost. Which of these approaches will, thus, progress towards practical products still remains to be seen in the future, however, it is likely that more attention should be given in the future to the approaches that can provide environmentally stable and cost-efficient designs. As rotation sensors are the workhorse of modern mechanical measurement systems, this field will likely see continued research interest, both in academia and industry in the future.
